# Purple Urine Bag Syndrome: Antibiotic Treatment or Not?

**DOI:** 10.7759/cureus.86725

**Published:** 2025-06-25

**Authors:** Gunnhild Helmsdal

**Affiliations:** 1 Department of Health Research, National Hospital of the Faroe Islands, Tórshavn, FRO

**Keywords:** antibiotic resistance, indwelling catheter, pubs, purple urine bag syndrome, urinary tract infection

## Abstract

Purple urine bag syndrome (PUBS) is a relatively rare phenomenon characterized by purple discoloration of the urine, catheter, and collection bag in individuals with indwelling catheters often colonized by specific bacteria. Although typically asymptomatic, it requires appropriate diagnostic evaluation and, in some cases, targeted treatment. We present a case study of an 81-year-old male with an indwelling urinary catheter who developed an uncomplicated, self-limiting case of purple urine bag syndrome. The patient exhibited the characteristic purple discoloration of the catheter tubing and collection bag, without accompanying systemic symptoms such as fever, dysuria, flank pain, or altered mental status. This case underscores the importance of recognizing PUBS as a benign condition in many instances, to avoid unnecessary diagnostic testing or antibiotic therapy, while still ensuring appropriate clinical evaluation to rule out potential underlying infections or contributing risk factors.

## Introduction

Discoloration of the urine can be seen due to many different causes, resulting in a wide spectrum of colors: red, orange, purple, brown, black, white, blue, and green. The etiology of these discolorations can include underlying medical conditions, hemolysis, medications, foods, and supplements [1-3].

Purple discoloration is a rarer clinical entity and can sometimes be seen in acute porphyrias due to the accumulation and oxidation of porphyrin precursors, which darken the urine upon exposure to light and air [4]. Purple urine can also occur as a rare adverse effect of certain medications [5].

More commonly, purple urine bag syndrome (PUBS) is an uncommon condition characterized by a distinct purple discoloration of the urine due to particular bacteria causing urinary tract infections. PUBS was first described in 1978 [6]. The literature on this condition was limited for decades, but over the past 15 years, knowledge has increased. A 2019 literature review described PUBS as most commonly occurring in patients with long-term indwelling urinary catheters, and it has been associated with risk factors, including advanced age, female sex, constipation, immobility, and dementia [7].

It is difficult to estimate the prevalence of PUBS because most of the literature is based on case studies. The underlying mechanism involves the bacterial degradation of the essential amino acid tryptophan in the gut into indole, which is absorbed into the portal circulation. In the liver, indole undergoes conjugation and sulfation and is then excreted in the urine (indoxyl sulfate). In the urinary tract, bacteria such as *Escherichia coli*,* Proteus*,* Klebsiella*,* Enterococcus*,* Pseudomonas*, and *Providencia* species produce sulfatases and phosphatases that convert these compounds into the pigments indirubin (red) and indigo (blue), which combine to produce the characteristic purple color [6,7].

The discoloration can also appear without bacteria in the urine. Constipation leads to increased gut metabolism of tryptophan, chronic kidney disease can lead to increased levels of indoxyl sulfate, and spontaneous oxidation of the pigments can be promoted in alkaline urine. Also, the reaction between the pigments and the plastic (typically polyvinyl chloride (PVC)) of the catheter bag or tube can happen without discoloration of the urine itself [7].

The condition is usually easy to recognize clinically due to the vivid color. Patients are often asymptomatic, although careful evaluation is recommended as PUBS is frequently, though not always, associated with urinary tract infections [7]. Despite this, most patients receive antibiotic treatment, contributing to the global burden of antibiotic resistance. Here, we present a case of PUBS that illustrates that antibiotic therapy is not necessarily required initially, or even at all.

## Case presentation

The patient was an 81-year-old male with chronic hypertension, coronary heart disease, atrial fibrillation, hyperthyroidism, chronic kidney disease (CKD), and type 2 diabetes. His medications included Enalapril, Amlodipine, Metoprolol, Rosuvastatin, Nicorandil, Rivaroxaban, Levothyroxine, Metformin, and Dapagliflozin. His hypertension was well-regulated, he had no angina, his CKD was stable with eGFR 45, and the most recent glycated hemoglobin (HbA1c) was 34 mmol/mol. His chronic conditions were managed by his general practitioner, and he had no recent contact with the hospital sector. He was married, lived independently with his spouse, and did not receive home care services. He had a chronic indwelling urinary catheter, due to benign prostatic hyperplasia, for several years, and he did not experience recurrent urinary tract infections. His catheter was routinely changed every 10 weeks by his general practitioner.

**Figure 1:**
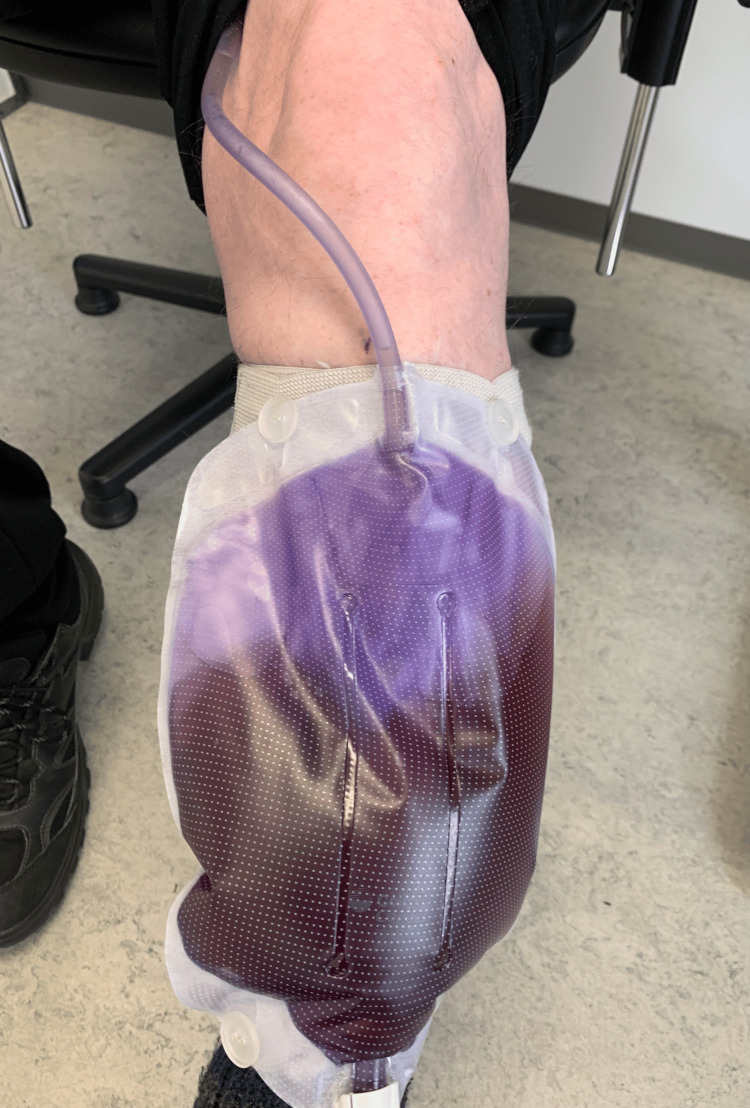
Dark brown urine and purple discoloration of the urine bag and catheter

He had noticed a change in the color of his urine and contacted his general practitioner. The patient did not report any other symptoms from the urinary tract, had no fever, and had a normal mental status. Upon examination, the urine appeared dark brown, while the catheter and urine bag were purple (Figure 1). A urine dipstick test was positive for leukocyte esterase, ketones, protein, and nitrite, and negative for erythrocytes (blood), with a slightly alkaline pH (Table 1).

**Table 1:** Urine analysis using a dipstick test. The first analysis was conducted on the day the patient contacted the general practitioner; the second was performed two days later. * The patient had type 2 diabetes and was being treated with Metformin and Dapagliflozin, the latter causing chronic glucosuria. ** Normal pH range: 4.6–8.0 (median: 6.0).

	PUBS	Post-PUBS
Leukocyt esterase	+++	+++
Nitrite	+	negative
Erythrocytes	negative	negative
Protein	+	negative
Ketones	+	negative
Glucose^*^	+++++	+++++
pH^**^	7.5	6.0

**Figure 2:**
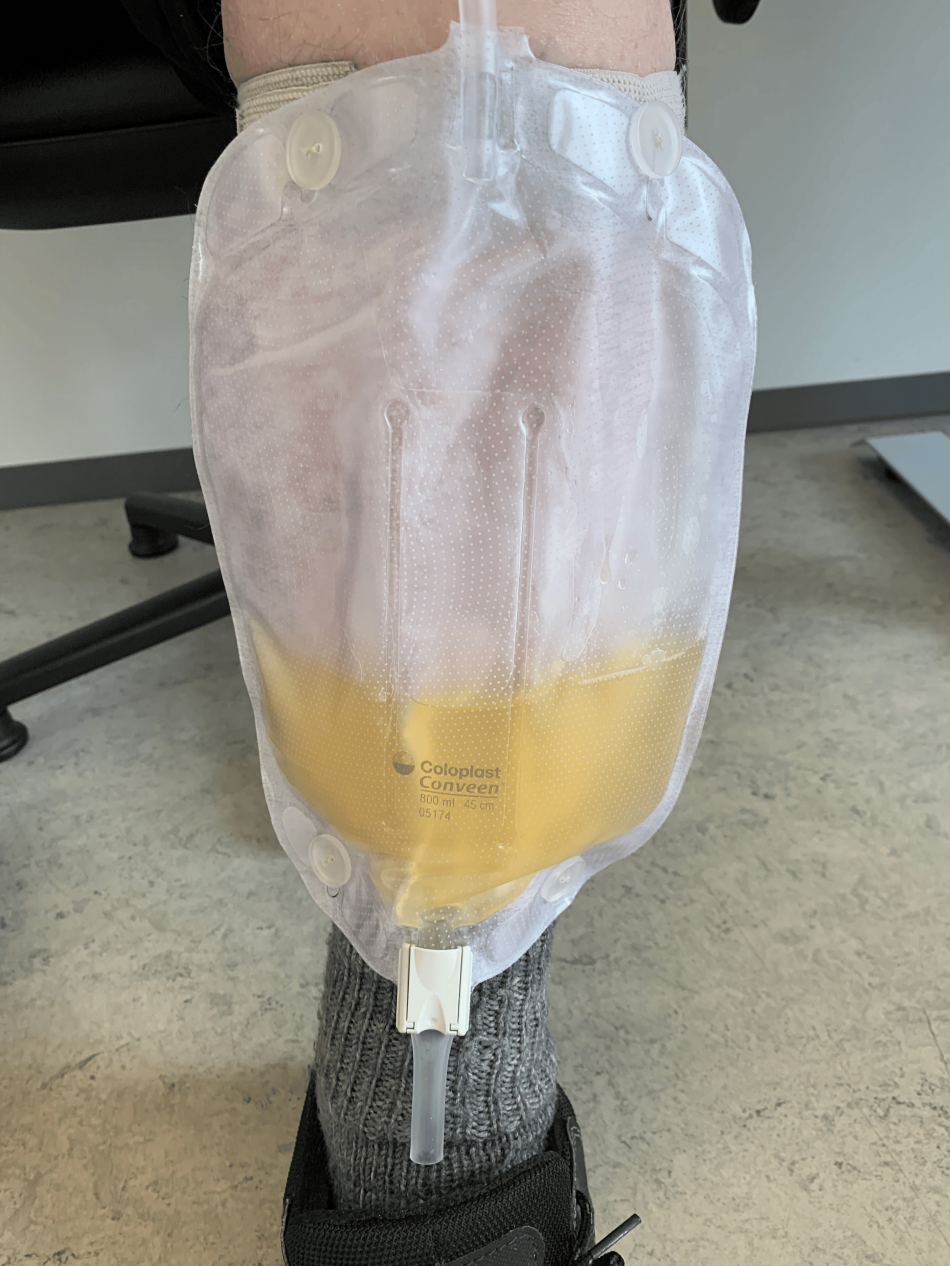
After changing the catheter and urine bag, the color of the urine normalized in less than a day

The urinary catheter was replaced, and the patient did not receive antibiotic treatment in accordance with the clinical recommendations of the Danish College of General Practitioners [8]. The urine color returned to normal approximately 12 hours later (Figure 2). Two days later, a urine culture revealed the growth of *Escherichia coli (E. coli)* at 10,000 CFU/mL, sensitive to all antibiotics in the local standard panel (Ampicillin, Mecillinam, Trimethoprim, and Sulfamethoxazole). A follow-up dipstick test on the same day confirmed the spontaneous improvement of the condition, except for persistent leukocyturia, which is a common finding in patients with chronic indwelling urine catheters due to local irritation (Table 1).

## Discussion

Urinary tract infections place a significant burden on healthcare systems worldwide, particularly complex cases, such as catheter-associated urinary tract infections, due to their high incidence, prevalence, antibiotic resistance, and associated mortality [9]. Asymptomatic bacteriuria is especially common in individuals with permanent indwelling catheters, largely due to the ascending colonization of bacteria that form biofilms along the catheter wall [10]. This frequently leads to the inappropriate use of urine cultures and, consequently, unnecessary antibiotic treatment, contributing not only to patient discomfort but, more critically, to the escalating problem of antibiotic resistance [11]. Recent interventions targeting healthcare providers responsible for diagnosing and managing catheterized patients have led to a notable reduction in the overtreatment of asymptomatic bacteriuria [12]. Current clinical guidelines in both the US and Europe are informed by such evidence and are intended for physicians across all specialties who provide direct patient care, with particular attention to hospital and long-term care settings [13,14].

Since patients with PUBS are frequently asymptomatic, management should follow existing guidelines for asymptomatic bacteriuria, which recommend against routine antibiotic treatment in the absence of symptoms. Several non-antibiotic interventions target the underlying conditions that predispose to bacteriuria and pigment accumulation:

Catheter hygiene: Regular replacement of catheter tubing and collection bags helps prevent biofilm formation on the plastic surfaces, thereby minimizing bacterial colonization.

Adequate hydration: Ensuring sufficient fluid intake dilutes the urine, reducing bacterial concentration and slowing pigment accumulation.

Management of constipation: Constipation enhances tryptophan metabolism in the gut, increasing indoxyl sulfate production. Laxatives may be indicated to reduce this effect.

Medication review: Certain medications can alkalinize the urine, which promotes pigment formation; reviewing and adjusting such medications may be beneficial.

Close clinical monitoring: Patients should be observed for any emerging signs of symptomatic urinary tract infection (e.g., fever, dysuria, altered mental status), which would warrant antibiotic therapy.

Despite these recommendations, much of the published literature on PUBS consists of case reports, where antibiotic therapy is often administered in both initial and follow-up settings. However, some reports clearly demonstrate that antibiotic treatment is not always necessary, reinforcing the importance of differentiating asymptomatic bacteriuria from symptomatic infection in this context [15-17].

The present case of PUBS represents a typical example of asymptomatic bacteriuria in an elderly patient with a long-term indwelling catheter.

While the condition may appear alarming due to the discoloration of urine and presence of bacteria, it typically does not require antibiotic treatment. As demonstrated, management primarily involves catheter replacement, with antibiotics reserved only for patients who exhibit clinically significant symptoms. Although severe infections associated with PUBS have been documented [18,19], they are rare and generally occur alongside systemic symptoms. This case underscores the benign nature of PUBS in the absence of such symptoms and highlights the importance of thorough clinical assessment to prevent unnecessary medical interventions.

## Conclusions

As the condition purple urine bag syndrome (PUBS) is relatively rare and the presentation is somewhat unusual, the purple discoloration seen in PUBS often causes concern among patients and caregivers. It is therefore important to recognize the condition, perform the appropriate diagnostic tests, and initiate treatment that minimizes unnecessary antibiotic use and the risk of resistance. If antibiotic treatment is considered, therapy should be guided by local antibiotic stewardship protocols and susceptibility patterns.

In developing countries with limited access to health care, including access to trained professionals, appropriate diagnostics, and follow-up, this approach could be problematic or even impossible. However, it is still of paramount importance to educate primary sector caregivers about the condition and adhere to protective measures such as preventing constipation, ensuring proper hydration, maintaining good catheter hygiene, and regularly changing the catheter and collection bags. 
